# Empirical validation of the “Pediatric Asthma Hospitalization Rate” indicator

**DOI:** 10.1186/1824-7288-40-7

**Published:** 2014-01-21

**Authors:** Lorenza Luciano, Jacopo Lenzi, Kathryn Mack McDonald, Simona Rosa, Gianfranco Damiani, Giovanni Corsello, Maria Pia Fantini

**Affiliations:** 1 Department of Biomedical and Neuromotor Sciences, Alma Mater Studiorum, University of Bologna, Via San Giacomo 12, Bologna 40126, Italy; 2 Center for Health Policy/Center for Primary Care and Outcomes Research, Stanford University, Stanford, USA; 3 Institute of Hygiene, Catholic University of the Sacred Heart, Rome, Italy; 4 Department of Sciences for Health Promotion and Mother and Child Care, University of Palermo, Palermo, Italy

**Keywords:** Health services research, Quality of care, Quality indicators, Pediatrics, Asthma

## Abstract

**Background:**

Quality assessment in pediatric care has recently gained momentum. Although many of the approaches to indicator development are similar regardless of the population of interest, few nationwide sets of indicators specifically designed for assessment of primary care of children exist. We performed an empirical analysis of the validity of “Pediatric Asthma Hospitalization Rate” indicator under the assumption that lower admission rates are associated with better performance of primary health care.

**Methods:**

The validity of “Pediatric Asthma Hospitalization Rate” indicator proposed by the Agency for Healthcare Research and Quality in the Italian context was investigated with a focus on selection of diagnostic codes, hospitalization type, and risk adjustment. Seasonality and regional variability of hospitalization rates for asthma were analyzed for Italian children aged 2–17 years discharged between January 1, 2009, and December 31, 2011 using the hospital discharge records database. Specific rates were computed for age classes: 2–4, 5–9, 10–14, 15–17 years.

**Results:**

In the years 2009–2011 the number of pediatric hospitalizations for asthma was 14,389 (average annual rate: 0.52 per 1,000) with a large variability across regions. In children aged 2–4 years, the risk of hospitalization for asthma was 14 times higher than in adolescents, then it dropped to 4 in 5- to 9-year-olds and to 1.1 in 10- to 14-year-olds. The inclusion of diagnoses of bronchitis revealed that asthma and bronchitis are equally represented as causes of hospital admissions and have a similar seasonality in preschool children, while older age groups experience hospital admissions mainly in spring and fall, this pattern being consistent with a diagnosis of atopic asthma. Rates of day hospital admissions for asthma were up to 5 times higher than the national average in Liguria and some Southern regions, and close to zero in some Northern regions.

**Conclusions:**

The patterns of hospitalization for pediatric asthma in Italy showed that at least two different indicators are needed to measure accurately the quality of care provided to children. The candidate indicators should also include day hospital admissions to better assess accessibility. Future evaluation by a structured clinical panel review at the national level might be helpful to refine indicator definitions and risk groupings, to determine appropriate application for such measures, and to make recommendations to policy makers.

## Background

In recent years, there has been increasing awareness and concern about the care of children and adolescents at the hospital and primary-care level. A recent paper published in The Lancet underscored that Western European health care systems are not responsive to changes in children’s health needs: noncommunicable diseases are becoming increasingly common causes of childhood illness, and although overall children’s health has improved throughout Europe, large disparities persist [1]. Attention to quality assessment in pediatrics is increasing because indicators that proved to be valid for adult populations are not suitable for children and adolescent populations.

As regards primary health care quality, in 1993 Billings [2] first presented a list of ambulatory care sensitive conditions (ACSCs), i.e. conditions for which it is possible to prevent acute exacerbations and reduce the need for hospital admission through timely and effective interventions from primary health care (PHC) [3]. The most common ACSCs for children include asthma, gastroenteritis and urinary infections. When PHC services fail to ensure continuity of care with hospital care and specialty services, frequent and unnecessary hospitalizations for these conditions can result in fragmented pediatric care, as often reported by the families [4], and in expensiveness of health care services.

The Agency for Healthcare Research and Quality (AHRQ) in the United States emphasizes the need for consensus on a standardized methodology to develop a set of pediatric ACSC indicators. This process needs to be built on a serial and iterative methodology and should be supported by health care researchers, pediatricians and policy makers [5,6]. In assessing quality of children’s care, AHRQ has disseminated a software and specification tool with area level indicators measuring access to outpatient care and good management of ACSCs [7].

Recent publications have emphasized the need to review carefully the selection of diagnoses to be included in ACSC indicators when these hospitalizations are intended to measure the performance of PHC [8]; moreover, candidate indicators should be adapted to the context of the specific health care system, that can influence the relation between ACSC indicators and PHC. According to the AHRQ [9], the development of such indicators requires, after a literature review, a series of empirical analyses (i.e. investigating alternative definitions of the indicators) in order to establish their validity, detect possible bias and design appropriate risk adjustment models before submitting candidate indicators to clinical panelists.

In Italy, some outcomes indicators have been proposed by the National Outcomes Program (*Programma Nazionale Esiti* [PNE]) run by the National Agency for Regional Healthcare Services (Age.Na.S.) and the Performance Evaluation System implemented by the Tuscany region in order to set up a national model for hospital and PHC performance evaluation based on data routinely collected in electronic administrative databases [10,11].

In this set of measures, asthma is included among ACSCs as a major clinical concern in childhood due to its substantial burden on families and health care services. Moreover, a number of studies have shown an association between higher asthma hospitalization rates and low-quality processes of outpatient care—i.e. low use of inhaled anti-inflammatory agents and oral steroids [12], not having a written asthma management plan [13], and lack of continuity of care with the same provider [14].

Following the AHRQ process for quality indicator validation [9], the aim of this study is to perform an empirical analysis on the validity of “Pediatric Asthma Hospitalization Rate” indicator in the Italian context under the assumption that lower admission rates for one of the most common chronic conditions in children are associated with better performance of PHC, increased accessibility to outpatient services and overall better quality of care for children.

## Methods

### Context

Italy has a National Health Care System (NHCS) that provides uniform and comprehensive care. Under the Italian Constitution and its recent modification (2001), the State has exclusive power to set the ‘essential levels of care’ for all residents throughout the country, while the regions have responsibility for the organization and administration of publicly financed health care.

The hospital sector has long dominated the health care system in Italy. Inpatient care accounts for 48% of total public health care expenditure nationally and in some regions for almost 54%. Official policy for many years was to reduce NHCS bed capacity; in addition, recent reforms were aimed at reducing the use of hospital care [15] and enhancing PHC.

PHC is provided by general practitioners and family pediatricians remunerated on a capitation base/formula; they are in charge of providing care and assess patients needs, order diagnostic procedures, prescribe drugs, and refer patients to specialists and hospitals. They thus act as ‘gatekeepers’ for the system and ensure continuity of care to patients with chronic diseases.

Children aged 0–14 years^a^ are required to register to a family pediatrician. Differently from general practitioners with expertise or interest in pediatrics, family pediatricians are trained specialists who work at the primary-care level [16] providing ambulatory and home care, coordination of care for chronically ill patients and consults with subspecialties during weekdays working hours.

### Data

We explored the key elements of “Pediatric Asthma Hospitalization Rate” indicator through empirical analyses, as recommended by the AHRQ. In particular, we analyzed:

(1) Diagnostic codes selection;

(2) Hospitalization type: daytime hospital care (‘day hospital admissions’)^b^*vs*. ordinary hospitalizations;

(3) Need for risk adjustment.

The data source was the Italian hospital discharge records database (HDRs, in Italian *Schede di Dimissione Ospedaliera*). Information contained in HDRs is transmitted by all public and private hospitals to their own region, and every six months from the region to the Ministry of Health. Since 1995, all HDRs have been entered in a Hospital Information System database. The database includes demographic characteristics, admission and discharge dates, admission referral source, discharge status, principal diagnosis, up to five secondary diagnoses, and up to six interventions. The HDR-DRG (Diagnosis Related Group) system is systematically used to allocate funds to hospitals and to monitor quality of care and outcomes.

The population of interest included all patients aged 2–17 years discharged between January 1, 2009 and December 31, 2011 with a diagnosis of asthma (ICD-9-CM code: 493.xx). We also analyzed children with a primary diagnosis of bronchitis [466.0 (acute bronchitis), 490 (bronchitis, not specified as acute or chronic), 491.xx (chronic bronchitis)]^c^. This population includes all children potentially eligible for one of the asthma hospitalization rates currently in use. The lower age limit was set to 2 years, because evidence that in younger children asthma (or more broadly wheezing) is a chronic and preventable condition is lacking [17].

Records were excluded from the analysis if the following criteria were met:

(1) Major Diagnostic Category code 14 (pregnancy, childbirth and puerperium);

(2) Transfers from other hospitals or intermediate health care facilities;

(3) A diagnostic code of cystic fibrosis (277.0x) or anomalies of the respiratory system (747.21, 748.3–748.61, 748.69, 748.8, 748.9, 750.3, 759.3, 770.7).

### Statistical analyses

Pediatric admission rates were calculated as the average annual number of hospitalizations over the population aged 2–17 years per 1,000 inhabitants. Specific hospitalization rates were calculated for the following age classes: 2–4, 5–9, 10–14, 15–17. Age-adjusted rates were computed using the Italian population on January 1, 2002 as the standard population.

The seasonality and the regional variability of hospitalization rates were examined using Stata software, version 13 (StataCorp. 2013. *Stata Statistical Software*: *Release 13*. College Station, TX: StataCorp LP).

### Ethics statement

The study was carried out in compliance with the Italian law on privacy (Art. 20–21, DL 196/2003). Data were encrypted at the regional statistical offices where a unique identifier was assigned to each patient. This identifier does not allow tracing the patient’s identity and other sensitive data. When encrypted administrative data are used to inform health care planning activities, studies are exempt from notification to the Ethics Committee and no specific written consent is needed to use patient information stored in the hospital databases.

## Results

Over the study period (2009–11) the number of pediatric hospitalizations for asthma (2–17 years) was 14,389 (average annual rate: 0.52 per 1,000). Table 1 shows the hospital admission rates stratified by age group and the relative risks in children at different ages compared with adolescents. In 2–4 age group, the risk of hospitalization for asthma was 14 times higher than in adolescents, then it dropped to 4 in 5- to 9-year-olds and to 1.1 in 10- to 14-year-olds.

**Table 1:** **Asthma hospitalizations in Italy by age group** (**years 2009**–**11**)

**Age group**	**Number of hospitalizations**	**Average annual rate (per 1,000)**	**RR**
2–4 years	7,742	1.50	15.0
5–9 years	4,319	0.50	5.0
10–14 years	1,815	0.21	2.1
15–17 years	513	0.10	Ref.
*Total*	*14*,*389*	*0.52*	

RR, risk ratio.

*Data source*: Ministry of Health.

**Figure 1:**
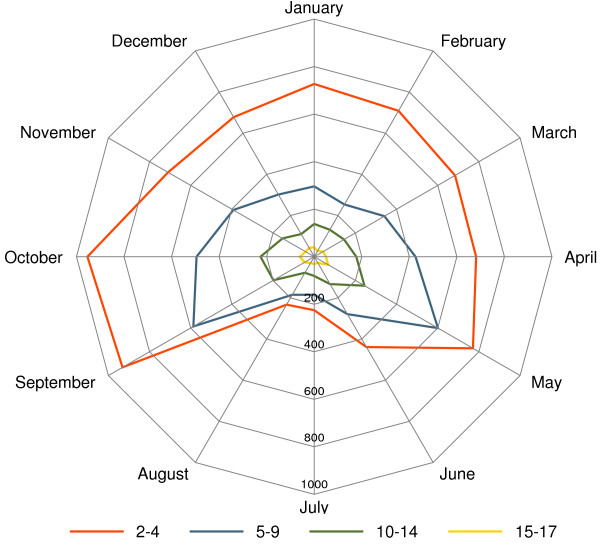
**Radar chart of monthly hospital admissions for asthma by age group in Italy.***Note*: All figures include only ordinary hospitalizations, except where otherwise specified. *Data source*: Ministry of Health.

The seasonality of hospital admissions by age group is presented in Figure 1. The 2–4 age group was hospitalized mainly in winter, as well as in early spring, September and October. Starting from school age, the seasonal pattern was very similar in all age groups, the highest number of hospitalizations being registered in May, September and October.

**Figure 2:**
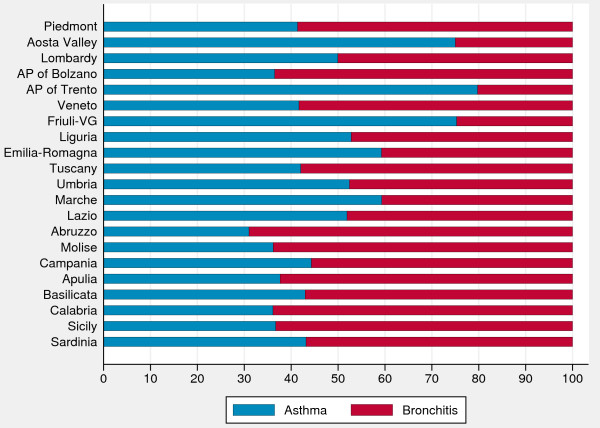
**Percentages of hospital admissions for asthma and bronchitis by region (2**–**4 years).** AP, autonomous province; VG, Venezia Giulia. *Data source*: Ministry of Health.

The peculiar seasonality of asthma hospitalizations among 2- to 4-year-old children led us to analyze the relative contribution of hospitalizations for asthma and bronchitis, and to explore the variability in diagnostic coding among regions. Among preschool children (Figure 2), diagnoses of bronchitis were generally higher than asthma, while among children aged at least 5 years (see Additional file 1: Figure S1) asthma was more frequent than bronchitis with the exception of some Southern regions, where the percentages of asthma and bronchitis were almost the same.

**Figure 3:**
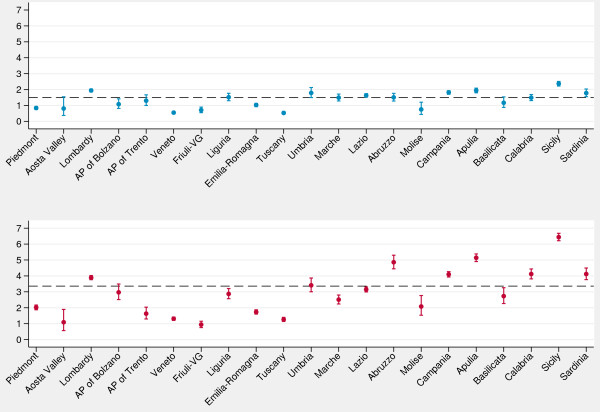
**Caterpillar plots of regional admission rates (per 1,000) for asthma (blue) and for asthma and bronchitis (red) (2–4 years).***Note*: Dashed line, national average. *Data source*: Ministry of Health.

The inclusion of diagnoses of bronchitis affects the regional performance of the indicator compared with the national average. In particular, if we consider hospitalizations for both asthma and bronchitis in preschool children (Figure 3) the national rate strongly increases, as well as the variability of regional rates: in some Southern regions, asthma-and-bronchitis rates are almost three times higher than asthma rates. As for children aged 5 and up, the regional pattern of the two indicators is very similar (see Additional file 2: Figure S2).

**Figure 4:**
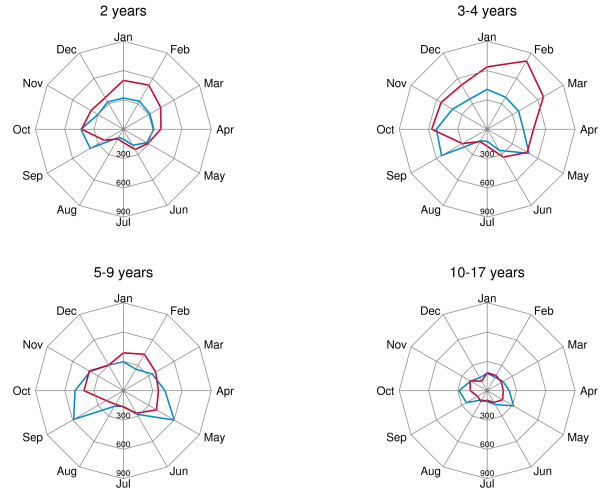
**Radar charts of monthly hospital admissions for asthma (blue) and bronchitis (red) (age groups: 2, 3–4, 5–9, 10–17).***Data source*: Ministry of Health.

Radar chart exploring seasonality of asthma and bronchitis admissions in children aged 2 years (Figure 4) shows that in this age group hospitalizations of asthma and bronchitis have a similar monthly distribution over the year, and both reach their peak in winter and early fall; results are very similar for 3- and 4-year-old children (Figure 4). On the contrary, starting from the age of 5, asthma and bronchitis show a different seasonality pattern (Figure 4).

**Figure 5:**
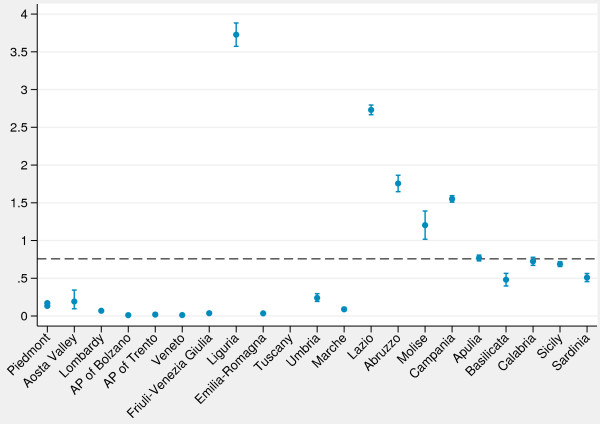
**Caterpillar plot of age**-**standardized rates (per 1,000) of day hospital admissions for asthma (2**–**17 years).***Note*: Dashed line, national average. *Data source*: Ministry of Health.

Lastly, we explored day hospital admissions for asthma. The caterpillar plot (Figure 5) reveals a huge variability among regions: in Liguria and some Southern regions the rate of day hospital admissions for asthma was even up to 5 times higher than the national average, while in some Northern regions rates were close to zero.

## Discussion

This study reports empirical analyses conducted on pediatric asthma admission rate as a candidate indicator of the quality of Italian PHC for this chronic condition.

As expected from a clinical point of view, we found that preschool children experienced more hospital admissions than school children and adolescents. A higher risk of hospitalization for a child in earlier age could be itself a demonstration of ‘known-group validity’ of the indicator, as previously reported in the US pediatric population [5].

Beyond this consideration, we argue that a problem of misclassification/miscoding could affect the indicator, when only asthma codes are taken into account in preschool children. The high number of hospital admissions of younger children in winter months (January, February, March) compared with the opposite seasonal pattern at school ages (May, September and October) suggests two different clusters of pathologies: the first, more likely to be diagnosed in winter months, may encompass bronchitis and other respiratory infectious diseases; the latter, represented by asthma and allergic conditions, is more frequently diagnosed in spring or fall.

From a clinical point of view, in infants and preschool children it is not possible to make a diagnosis of asthma and is thus preferable to adopt the term ‘wheezing’ , a condition which encompasses a range of symptoms including breathlessness and whistling sounds [18-21]. Bronchospasm and subsequent wheezing might be triggered by respiratory infections, and this could explain the similar seasonality of asthma and bronchitis in younger children, as suggested by our data.

Among preschool children, asthma and bronchitis are equally represented as causes of hospital admissions and have a similar seasonal pattern. Therefore, we suggest to build a specific indicator for children aged 2–4 years which combines the diagnostic codes of asthma and bronchitis. We acknowledge, however, that problems of labels of diseases are relevant in preschool years and not easily solvable using administrative data, so this point needs to be discussed extensively by a clinical panel. Older age groups (5–9, 10–14, 15–17) experience hospital admissions mainly in spring and fall. This pattern might be consistent with a diagnosis of asthma, especially atopic asthma. As the risk of hospital admission decreases as age increases, we think that it would be fair to consider age-adjusted asthma hospitalization rate (5–17 years) when comparative analyses are needed.

Moreover, caterpillar plots stratified by age groups revealed that, although Southern regions have generally higher rates and Northern regions have lower rates, the regional performances differ among the age groups considered. These empirical findings suggest that age is not only a confounder, but also an effect modifier of regional performances, and stratification by age group (2–4 *vs*. ≥5 years) might enhance the clinical understanding of the phenomenon as well as the policy makers’ decision process.

Lastly, our results show that day hospital care for asthma varied considerably among regions. It is possible that in some regions day hospital admissions are inappropriately used to complete a diagnostic workup that could be performed in ambulatory settings, confirming that in our country this is an important issue to be considered when inappropriate use of hospital beds and avoidable admissions are concerned.

Our results should be interpreted keeping in mind that administrative data rely on the accuracy and completeness of patients’ charts, as well as of ICD-9-CM coding. However, HDRs allow answering health services research questions that would be impractical or impossible to address in clinical studies [22].

## Conclusions

In the process of adaptation of the asthma admission rate indicator to the Italian context, our findings point to the need to consider at least two different indicators of preventable asthma hospitalizations, one for children aged 2 to 4 years (diagnoses of asthma and bronchitis combined) and another for school children and adolescents (only diagnosis of asthma). Another key finding of our study is that the indicators should include day hospital admissions because these admissions may be important to define appropriate hospital utilization.

Future evaluation of these analyses by a structured clinical panel review involving specialists from the pediatric field at the regional and national level might be helpful to refine indicator definitions and risk groupings, to determine appropriate application for such measures, and make recommendations to policy makers regarding the best indicators for inclusion in a national pediatric indicator set. Using definitions aligned with those in use by other countries would make international comparison more meaningful. However, tailoring indicators to the local Italian context would offer useful tools to clinicians and policy makers interested in improving pediatric care locally. By conducting detailed empirical analyses on alternative definitions within Italy, clinical panels could consider the tradeoffs between more tailored versus international definitions. Finally, this series of analyses offers a template for researchers from other countries to examine patterns at national and regional levels in order to understand the potential influence of coding variation, referral practices, and seasonality by specific age groups. Such application of indicator components is a first step on the path to a closer look at ways to monitor children’s care and identify aspects amenable to delivery systems and policy improvements.

### Endnotes

^a^The pediatrician is compulsory for children aged up to 6 years, and for children aged 6 to 14 years the choice may be between a pediatrician and a general practitioner. Under certain circumstances (chronic conditions or special health needs) the age limit is extended up to 16 years.

^b^In Italy, daytime hospital care (day hospital) consists in a planned one-day admission to the hospital without overnight stay, to perform diagnostic procedures and/or surgical, therapeutic or rehabilitative care. These diagnostic and therapeutic procedures require specific instrumental techniques, are carried out by multidisciplinary staff and are more complex than those run on an outpatient basis.

^c^We did not include diagnoses of wheezing in the indicator (ICD-9-CM code 786.07) because, over the study period, only 177 pediatric hospitalizations had this code. Still, there was evidence of an increasing trend in wheezing diagnoses between 2009 and 2011 (28 to 93 cases), suggesting that the inclusion of this code in further indicator updates should be considered.

## Abbreviations

ACSC: Ambulatory care sensitive condition; PHC: Primary health care; AHRQ: Agency for Healthcare Research and Quality; PNE: Programma Nazionale Esiti; Age.NA.S.: National Agency for Regional Healthcare Services; NHCS: National Health Care System; HDR: Hospital Discharge Record; DRG: Diagnosis Related Group; ICD-9-CM: International classification of diseases (Clinical Modification, Ninth Revision); RR: Risk ratio; AP: Autonomous Province; VG: Venezia Giulia.

## Competing interests

The authors declare that they have no competing interests.

## Authors’ contributions

LL contributed to the conception of this paper, conceived the study design and drafted the manuscript; JL conceived the statistical methodology, performed the statistical analysis and drafted the manuscript; KMM participated in the study design and helped to draft the manuscript; SR took responsibility for the integrity of the data and performed the statistical analysis; GD provided data sources and participated in the study design; GC critically revised the draft and contributed to the final writing of the paper; MPF contributed to the conception of this paper, conceived the study design and drafted the manuscript. All authors read and approved the final version of the manuscript.

## Supplementary Material

Additional file 1: Figure S1 Percentages of hospital admissions for asthma and bronchitis by region (5–17 years). *Data source*: Ministry of Health. Click here for file

Additional file 2: Figure S2 Caterpillar plots of age-standardized regional admission rates (per 1,000) for asthma (blue) and for asthma and bronchitis (red) (5–17 years). *Note*: Dashed line, national average.*Data source*: Ministry of Health. Click here for file
