# Supplementary figures and images for: Empirical validation of the “Pediatric Asthma Hospitalization Rate” indicator
Source: Ital J Pediatr. 2014 Jan 21;40:7. doi: 10.1186/1824-7288-40-7 (PMC3899920; doi:10.1186/1824-7288-40-7)

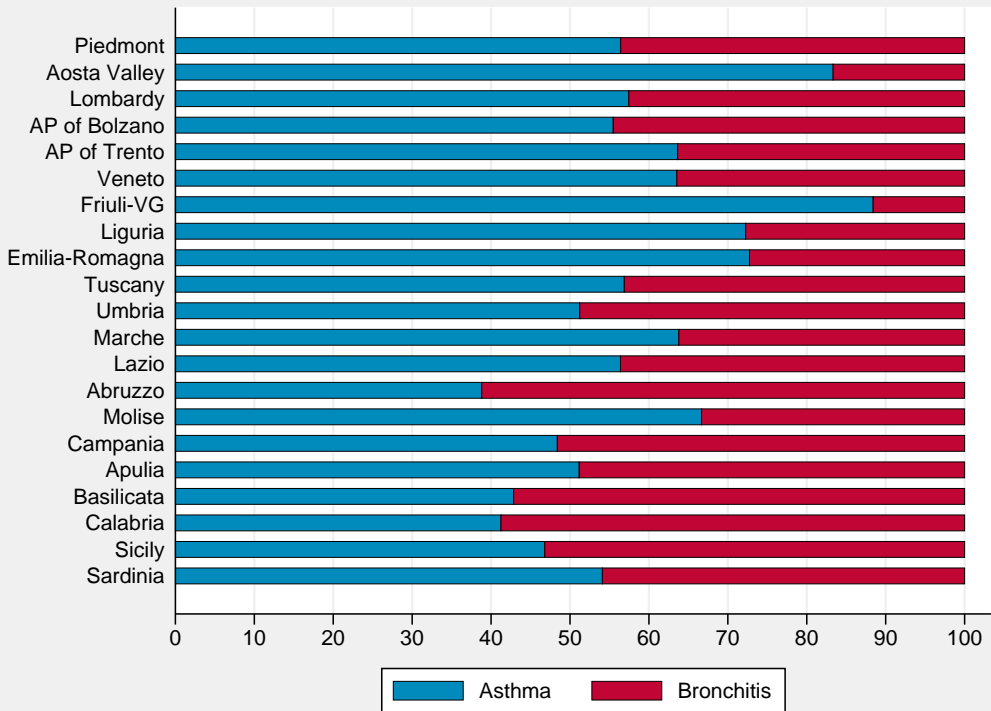

Supplement: Additional file 1: Figure S1 — Percentages of hospital admissions for asthma and bronchitis by region (5–17 years). Data source: Ministry of Health. [file 1824-7288-40-7-S1.pdf]

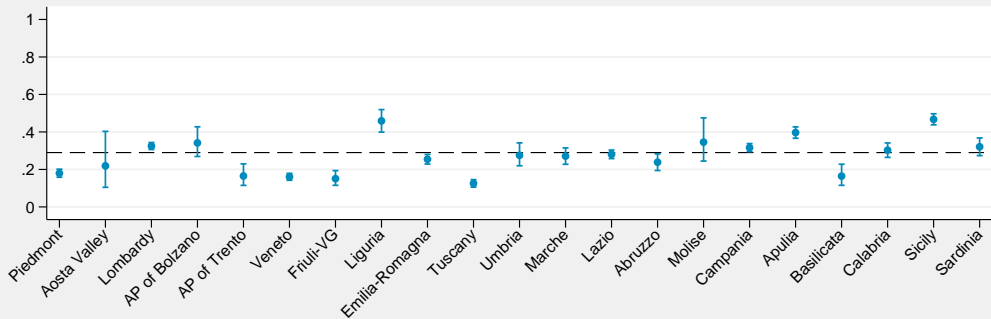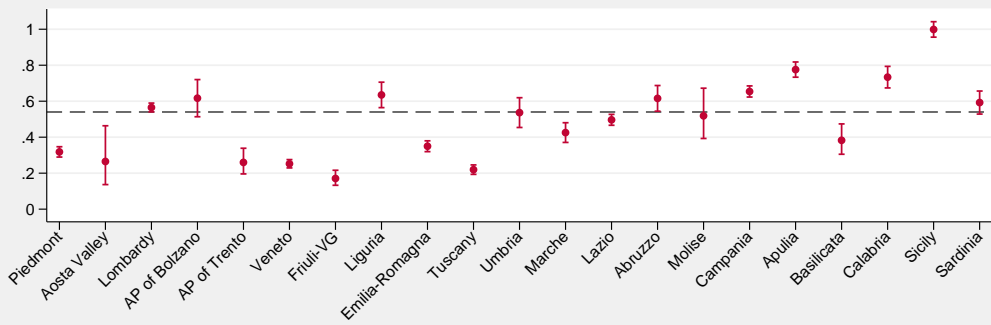

Supplement: Additional file 2: Figure S2 — Caterpillar plots of age-standardized regional admission rates (per 1,000) for asthma (blue) and for asthma and bronchitis (red) (5–17 years). Note: Dashed line, national average.Data source: Ministry of Health. [file 1824-7288-40-7-S2.pdf]
